# A Comparative Study of Changes in Huanglian Jiedu Decoction with Combined Decoction and Single Decoction Based on Metabolomics-Physical Characterization-Transcriptomics Correlation Analysis

**DOI:** 10.3390/ph18121815

**Published:** 2025-11-27

**Authors:** Yue Luan, Ruotong Lv, Qian Wang, Weiqi Wang, Yuanlu Zhang, Zhidong Qiu, Ye Qiu

**Affiliations:** School of Pharmaceutical Sciences, Changchun University of Chinese Medicine, Changchun 130117, China; 15843186923@163.com (Y.L.); 18946765753@163.com (R.L.); 17319605765@163.com (Q.W.); 18304201506@163.com (W.W.); 15844675576@163.com (Y.Z.); qzdcczy@163.com (Z.Q.)

**Keywords:** combined decoction, single decoction, chemical composition, physical indicators, transcriptome sequencing

## Abstract

**Background:** Using Huanglian Jiedu decoction (HJD) as a model, this study systematically compares the traditionally prepared combined decoction with mixtures of separately decocted components, focusing on differences in chemical composition, material properties, and transcriptomic responses. **Methods:** To maintain consistency, both the combined HJD decoction and the single-herb mixture were prepared using a standardized method, and their chemical profiles were analyzed by HPLC and UPLC-MS/MS to identify constituent differences. Physical properties were examined through key parameter measurements and phase behavior analysis, and the integration of chemical and physical data identified the components driving the observed material changes. Transcriptome sequencing compared the two decoction types, highlighting differentially expressed genes and the major regulatory pathways involved. **Results:** HPLC analysis showed a clear redistribution of components between the two decoction methods, with the combined decoction containing higher levels of alkaloids such as coptisine chloride, epiberberine, palmatine chloride, jatrorrhizine hydrochloride, and phellodendrine chloride, while the single decoction mixture had higher levels of berberine hydrochloride and baicalin. In the combined decoction, the berberine hydrochloride content was 37.04 mg/g, and the baicalin content was 15.57 mg/g; in the single decoction, the berberine hydrochloride content was 41.15 mg/g; in the combined decoction, the baicalin content was 40.07 mg/g. UPLC-MS/MS analysis confirmed clear differences between the two decoctions, mainly in flavonoid and alkaloid compositions. The combined decoction contains 110 flavonoid compounds and 67 alkaloid compounds, while the single decoction contains 100 flavonoid compounds and 80 alkaloid compounds. Physical measurements showed that the combined decoction had higher total dissolved solids, conductivity, and salinity, while the single decoction had higher resistivity. The combined decoction had a TDS of 2480 mg/L, σ of 4.95 ms/cm, S of 0.26%, and ρ of 202 Ω·cm; the single decoction had a TDS of 1190 mg/L, σ of 2.37 ms/cm, S of 0.12%, and ρ of 419 Ω·cm. Phase separation analysis indicated that the combined decoction formed a stable nanoscale phase structure, whereas the single decoction remained unstable. Transcriptome sequencing at various concentrations revealed marked differences in gene expression between the two preparations, reflecting their distinct biological activities. **Conclusions:** Analysis showed clear differences in chemical composition, physical properties, and gene expression in the combined decoction. Taking Huanglian Jiedu decoction as a representative example, we systematically compared the specific differences between combined and single decoction methods, providing a reference basis for subsequent pharmacodynamic evaluation and clinical application.

## 1. Introduction

Traditional Chinese herbal decoctions are a primary liquid dosage form prepared by combining medicinal slices and extracting them with water through soaking, boiling, and simmering, after which the residue is removed and the liquid is taken orally [1]. During preparation, active compounds are released into the water and can interact to produce synergistic effects that enhance therapeutic efficacy. These formulations are used to restore Yin–Yang balance, prevent disease progression, and treat various disorders. As a liquid form, the decoction allows rapid absorption and quick delivery of active ingredients to target sites, and its composition can be adjusted according to the patient’s condition, making it suitable for acute or severe cases [2].

Huanglian Jiedu decoction, a representative formula for clearing heat and detoxifying, consists of four herbs: Huanglian (dried rhizome of *Coptis chinensis Franch*, a plant of the Ranunculaceae family), Huangqin (dried root of *Scutellaria baicalensis Georgi*), Huangbo (dried bark of *Phellodendron chinensis Schneid*, a plant of the Rutaceae family), and Zhizi (dried mature fruit of *Gardenia jasminoides Ellis*, a plant of the Rubiaceae family). According to the traditional Chinese medicine principle of “sovereign, minister, assistant, and messenger,” Huanglian Jiedu decoction is formulated with *Coptis chinensis Franch* as the sovereign herb that clears heart fire; *Scutellaria baicalensis Georgi* as the minister herb that reduces lung heat; *Phellodendron chinense Schneid* bark as the assistant herb that alleviates kidney fire; and *Gardenia jasminoides Ellis* as the messenger herb that harmonizes and guides the overall action. Current research indicates that the core active compounds in Huanglian Jiedu decoction primarily include isoquinoline alkaloids from *Coptis chinensis Franch* and *Phellodendron chinensis Schneid* (represented by berberine, palmatine, and phellodendrine alkaloids), flavonoids from *Scutellaria baicalensis Georgi* (centered on baicalin and baicalein), cycloaromatic terpenoids from *Gardenia jasminoides Ellis* (primarily gardenin and genipin), and carotenoids (such as crocin) [3]. Among these, alkaloids and flavonoids constitute the highest proportion. Berberine, baicalin, and gardenin exhibit the most pronounced pharmacological effects, including antibacterial, anti-inflammatory, and antioxidant properties. These compounds form the core material basis for the formula’s efficacy in clearing heat and detoxifying, and are frequently used as key quality control markers [4,5]. Supported by long-standing clinical use, the multi-component structure of this formula enables a wide range of pharmacological effects through synergistic interactions. Contemporary research has confirmed that HJD exhibits antimicrobial, anti-inflammatory, metabolic regulatory, neuroprotective, and antitumor properties, among others [6,7,8,9,10]. Clinically, it is now applied in the management of various conditions such as tumors [11,12], arthritis [13,14], sepsis [15,16], cardiac injury [17], liver injury [18], cerebral ischemia [19], renal disorders [20], Alzheimer’s disease [21], type 2 diabetes [22], fungal infections [23], and inflammatory diseases [24].

According to traditional Chinese medicine formulation theory, herbal combinations function as integrated chemical systems where multiple interactions occur during co-decoction, leading to physicochemical reactions that can alter solubility and activity. Some components enhance the dissolution of active substances from other herbs, as seen when *Zingiber officinale Rosc* reduces the toxicity of Pinellia ternata during boiling, and new bioactive compounds may form through heat-induced reactions among different ingredients, which cannot be achieved by decocting each herb separately. During combined decoction, the main interactions between active components arise from non-covalent forces, such as hydrogen bonding [25,26,27], van der Waals forces, π-π stacking [28,29,30], hydrophobic interactions [31,32,33], coordination bonds [34], and electrostatic interactions [35,36,37]. Within compound decoctions, multiple chemical components (rather than a single chemical bond) spontaneously organize into structured systems, a process that drives alterations in their properties. Using Huanglian Jiedu decoction as a representative decoction, we systematically compared the specific differences in physical parameters and chemical constituents between combined and separate decoction methods, providing a reference basis for subsequent pharmacodynamic evaluation and clinical application.

## 2. Results

### 2.1. HPLC Comparison Results

**Figure 1:**
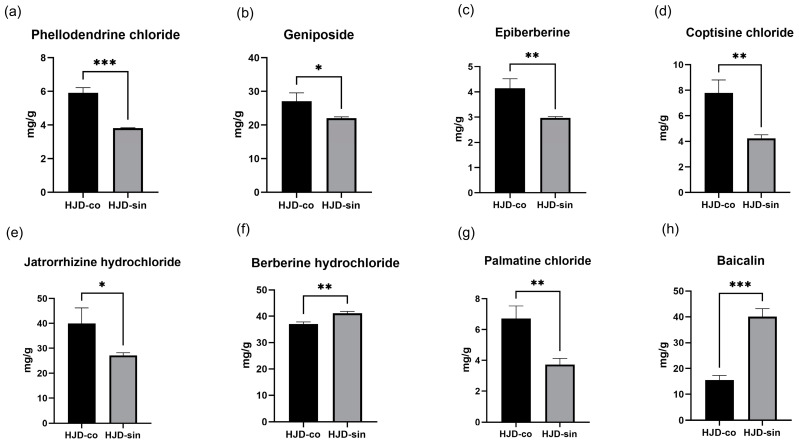
(**a**) Difference in phellodendrine chloride content between combined decoction and single decoction, (**b**) difference in geniposide content between combined decoction and single decoction, (**c**) difference in epiberberine content between combined decoction and single decoction, (**d**) difference in coptisine chloride content between combined decoction and single decoction, (**e**) difference in jatrorrhizine hydrochloride content between combined decoction and single decoction, (**f**) difference in berberine hydrochloride content between combined decoction and single decoction, (**g**) difference in palmatine chloride content between combined decoction and single decoction, (**h**) difference in baicalin content between combined decoction and single decoction. (* *p* < 0.05, ** *p* < 0.01, *** *p* < 0.001).

HPLC content analysis was conducted on eight indicator components of HJD, comparing the combined decoction with the single decoction. Calculations of the eight indicator components yielded the results shown in Figure 1. Six components—coptisine chloride, epiberberine, palmatine chloride, jatrorrhizine hydrochloride, phellodendrine chloride, and geniposide—were present at higher concentrations in the combined decoction. In contrast, berberine hydrochloride and baicalin exhibited greater abundance in the single decoction formulation. In the combined decoction, the berberine hydrochloride content was 37.04 mg/g, and the baicalin content was 15.57 mg/g; in the single decoction, the berberine hydrochloride content was 41.15 mg/g; and in the combined decoction, the baicalin content was 40.07 mg/g.

### 2.2. UPLC-MS/MS Comparison Results

**Figure 2:**
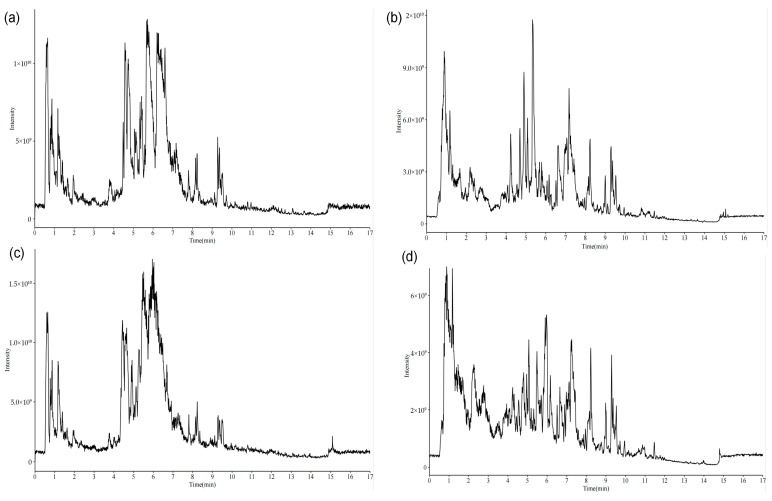
(**a**) Combined decoction positive ion chromatogram, (**b**) combined decoction negative ion chromatogram, (**c**) single decoction positive ion chromatogram, (**d**) single decoction negative ion chromatogram.

Non-targeted metabolomic profiling of both combined and single decoction samples was carried out by UPLC-MS/MS. The resulting total ion chromatograms, acquired in positive and negative ionization modes, are presented in Figure 2. Applying screening thresholds of peak area > 1 × 10^4^ and mass accuracy error < 5 × 10^−6^, a total of 583 compounds were detected in the combined decoction, compared to 588 in the single decoction.

**Figure 3:**
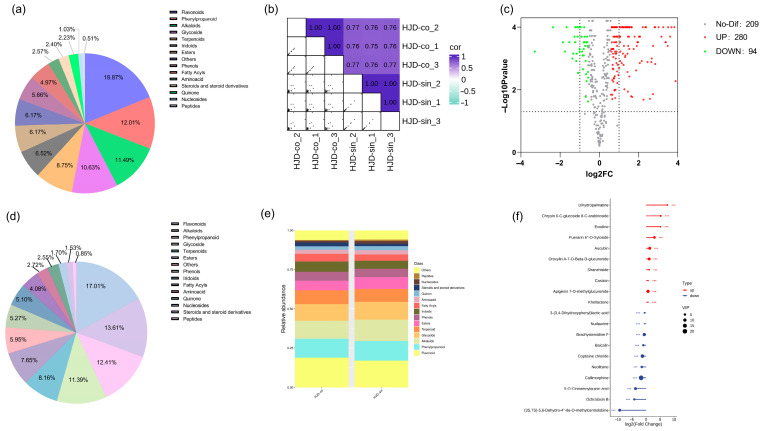
Panel (**a**) presents the compound classification profiles for the combined; (**b**) presents the compound classification profiles for the single decoctions; (**c**) shows a sample correlation heatmap; (**d**) provides a comparative bar chart of compound categories; (**e**) displays the volcano plot of differential metabolites; and (**f**) highlights metabolites with the most substantial fold-change differences. (*** *p* < 0.001).

Among these, the combined decoction contained 109 flavonoids, 67 alkaloids, and 70 phenylpropanoids, whereas the single preparation comprised 100 flavonoids, 80 alkaloids, and 73 phenylpropanoids. The distribution of additional compound categories is visualized in Figure 3a,d.

Calculations using Pearson’s correlation coefficient revealed high intra-group correlations among samples, while inter-group correlations varied in strength (Figure 3b), indicating potential differences in constituent compounds between groups. Figure 3e shows that flavonoids, alkaloids, phenylpropanoids, and terpenoids represent the predominant chemical classes in both decoctions. However, their relative abundances differ substantially depending on the preparation method.

Differential expression analysis was conducted to compare metabolite profiles between the combined and single decoction groups. Statistically significant and substantially altered compounds were identified by integrating both the *p*-value and fold change (FC) threshold, where FC denotes the ratio of the average abundance of each metabolite between the two groups. Metabolites meeting the criteria of FC > 1.5 or FC < 0.67 with a *p*-value < 0.05 were classified as differentially expressed.

Volcano plot visualization revealed 280 upregulated metabolites, 95 downregulated metabolites, and 208 non-significant metabolites in the combined decoction relative to the single decoction (Figure 3c). The top ten most significantly up- and down-regulated metabolites are displayed in Figure 3f. Among these differential metabolites, alkaloids and flavonoids were the most represented categories, with seven and six members, respectively, followed by cycloartenoids, phenylpropanoids, phenols, and others. These results suggest that the chemical distinctions between the two decoction methods lie mainly in flavonoids and alkaloids—the principal bioactive constituents of Huanglian Jiedu decoction [23,38,39,40,41,42].

#### Multivariate Statistical Analysis

**Figure 4:**
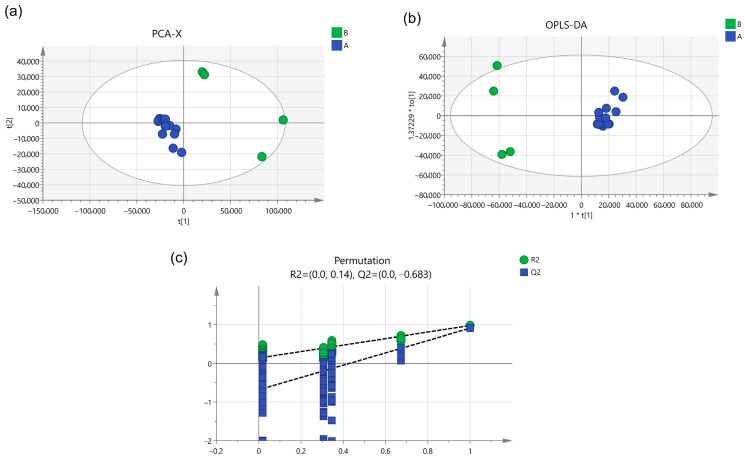
(**a**) PCA score plot, (**b**) OPLS-DA score plot, (**c**) permutation test plot.

To screen key compounds influencing the change in components during combined and single decoction, peak area data for the 20 most significantly different chemical components were normalized and imported into SIMCA14.1 software for PCA analysis. The results are shown in Figure 4a, where the data clustered into two distinct groups with Q^2^ = 0.998, indicating the model possesses excellent predictive capability. The PCA scoreplot revealed that Dihydropalmatine, Chrysin 6-C-glucoside 8-C-arabinoside, Evodine, Puerarin 6″-O-xyloside, Shanzhiside, Casticin, Apigenin 7-O-methylglucuronide, Khellactone, 3-(3,4-Dihydroxyphenyl)lactic acid, Nudaurine, Baicalin, Neolitsine, Callimorphine, 5-O-Cinnamoylquinic acid, Ochratoxin B, and (3S,7S)-5,6-Dehydro-4″-de-O-methylcentrolobine grouped together. Aucubin, Oroxylin A-7-O-Beta-D-glucuronide, Brachystemidine F, and Coptisine chloride also grouped together. In the PCA plot, distinct points represent different chromatographic peaks. Points farther from the origin indicate higher weight values and greater influence on the sample. Aucubin, Oroxylin A-7-O-Beta-D-glucuronide, Brachystemidine F, and Coptisine chloride are positioned farther from the origin, indicating these four components exert significant influence.

Subsequent OPLS-DA analysis was performed on the PCA data, with in-depth exploration of the 20 components. As shown in Figure 4b, R^2^X = 0.964, R^2^Y = 0.974, and Q^2^ = 0.909, demonstrating the model’s strong ability to distinguish between groups. To prevent overfitting in the OPLS-DA model that could compromise analytical accuracy, a permutation test was further conducted. Results are shown in Figure 4c: R^2^ = 0.14, Q^2^ = −0.683. The established model was verified as valid and reliable.

### 2.3. Comparison of Physical Parameters

**Figure 5:**
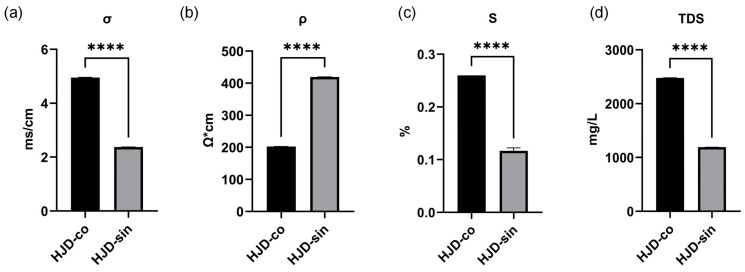
(**a**) Comparison chart of combined and single-stage conductivity (σ); (**b**) comparison chart of combined and single-stage resistivity (ρ); (**c**) comparison chart of combined and single-stage salinity (S); (**d**) comparison chart of combined and single-stage TDS. (**** *p* < 0.0001).

The conductivity of an aqueous solution is determined by its solute concentration. Higher ion levels increase charge density and ion migration rates, thereby elevating conductivity. Total dissolved solids (TDS), which reflect the concentration of ions like calcium, magnesium, sodium, and potassium, also influence this property: purer water shows lower TDS, reduced conductivity, and consequently higher resistivity [43]. In the case of Huanglian Jiedu decoction, the combined decoction had a TDS of 2480 mg/L, σ of 4.95 ms/cm, S of 0.26%, and ρ of 202 Ω·cm; the single decoction had a TDS of 1190 mg/L, σ of 2.37 ms/cm, S of 0.12%, and ρ of 419 Ω·cm. The combined preparation displayed noticeably greater TDS, conductivity, and salinity than the single decoction, whereas resistivity was lower (Figure 5). This indicates that the combined decoction contains more water-insoluble substances, resulting in significant differences in physical properties such as ionic strength and osmotic pressure compared to the single decoction.

### 2.4. Phase-Separation Morphological Analysis Results

#### 2.4.1. Results of the Tyndall Effect

**Figure 6:**
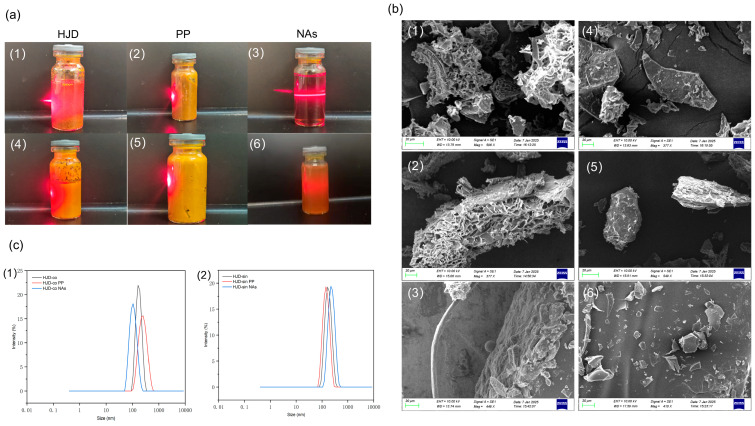
Panel (**a**) illustrates the Tyndall effect: (1) represents the HJD phase; (2) represents the HJD-co PP phase; (3) represents the HJD-co NAs phase; (4) represents the HJD-sin phase; (5) represents the HJD-sin PP phase; and (6) represents the HJD-sin NAs phase. SEM microstructural observations are shown in (**b**): (1) is an SEM image of the HJD phase; (2) is an SEM image of the HJD-co PP phase; (3) is an SEM image of the HJD-co NAs phase; (4) is an SEM image of the HJD-sin phase; (5) is an SEM image of the HJD-sin PP phase; and (6) is an SEM image of the HJD-sin NAs phase. Panel (**c**) presents particle size distributions, where (1) and (2) depict the results for HJD-co and HJD-sin.

For the combined decoction of Huanglian Jiedu decoction (HJD-co), the precipitate phase of the combined decoction (HJD-co PP), the nano-aggregates phase of the combined decoction (HJD-co NAs), the single decoction of Huanglian Jiedu decoction (HJD-sin), the precipitate phase of the single-decoction Huanglian Jiedu decoction (HJD-sin PP), and the nano-aggregates phase of the combined decoction Huanglian Jiedu decoction (HJD-sin NAs) were observed for the Tyndall effect, as shown in Figure 6a.

The results indicate that HJD-co and HJD-co PP liquids appear turbid with no clear light path, whereas HJD-co NAs liquid is clear with distinct light beams, exhibiting the Tyndall effect, confirming the formation of nanoaggregates in HJD-co NAs. The remaining HJD-sin, HJD-sin PP, and HJD-sin NAs liquids were all relatively turbid and did not exhibit the Tyndall effect.

#### 2.4.2. Results of the SEM Observation

Conduct microscopic morphological observations of various powdered samples of Huanglian Jiedu decoction using Scanning electron microscopy (SEM), including their precipitate and nano-aggregate phases (Figure 6b). The combined decoction (HJD-co) and its precipitate phase (HJD-co PP) displayed a regularly arranged network structure. Spherical particles with uniform size and shape were observed in the nano-aggregate phase of the combined decoction (HJD-co NAs). In contrast, the single decoction (HJD-sin) and its corresponding precipitate (HJD-sin PP) showed irregular, block-like morphologies. No such spherical particles were found in the nano-aggregate phase derived from the single decoction (HJD-sin NAs), which instead consisted of fragmented aggregates.

#### 2.4.3. Particle Size Distribution Results

Particle size distribution profiles for HJD-co and HJD-sin are presented in Figure 6c. Following phase separation, all fractions of the combined decoction (HJD-co) displayed sizes consistent with stable dispersion systems. The particle dimensions decreased in the order HJD-co PP > HJD-co > HJD-co NAs, where the nano-aggregate phase (HJD-co NAs) showed a predominant size distribution centered near 100 nm. By contrast, none of the single decoction (HJD-sin) phases exhibited a size profile meeting dispersion stability criteria, suggesting unsuccessful nanoparticle assembly and overall system instability.

### 2.5. Physical-Chemical Correlation Analysis

#### 2.5.1. Physical Parameters-Correlation Analysis of Component Types

**Figure 7:**
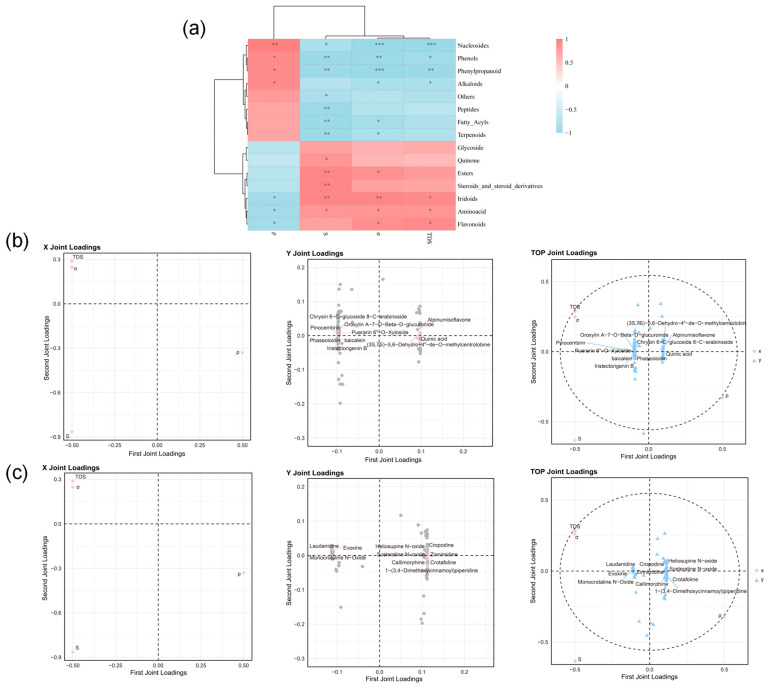
(**a**) Spearman correlation analysis among component types; (**b**) correlation between physical parameters and flavonoid constituents; (**c**) correlation between physical parameters and alkaloid constituents. (* *p* < 0.05, ** *p* < 0.01, *** *p* < 0.001).

Spearman’s rank correlation, a nonparametric statistical approach, assesses monotonic associations between variables without assuming linearity or normality in the data distribution. This method was applied to examine the relationship between standardized physical parameters and the relative expression levels of major chemical constituent categories, as illustrated in Figure 7a.

Total dissolved solids (TDS), conductivity (σ), and salinity (S) were positively correlated with several component classes—including flavonoids, amino acids, iridoids, esters, steroids and their derivatives, quinones, and glycosides—while showing negative correlations with other constituent groups. ρ showed strong correlations with Nucleosides, Phenols, Phenylpropanoids, and Alkaloids. This indicates that variations in most physical parameters correlate with the types of components present in the samples.

#### 2.5.2. Physical Parameters-Flavonoid Correlation Analysis

Orthogonal Projections to Latent Structures (O2PLS) is a multivariate analysis method that combines Principal Component Analysis (PCA) and Partial Least Squares (PLS) regression. It is effective for analyzing data with strong collinearity and interdependent variables. To clarify the relationship between physical parameters and flavonoid composition, a bidirectional O2PLS analysis was conducted linking physical measurements with the relative abundance of flavonoid compounds (Figure 7b).

Model reliability was evaluated using R^2^X and R^2^Y, yielding values of 0.999 and 0.964, indicating strong predictive performance. The analysis identified ten flavonoids most closely linked to variations in physical parameters, including (3S,7S)-5,6-Dehydro-4″-de-O-methylcentrolobine, Oroxylin A-7-O-β-D-glucuronide, Alpinumisoflavone, Chrysin 6-C-glucoside 8-C-arabinoside, 6″-O-Xyloside, Pinocembrin, Puerarin, Quinic acid, Baicalein, Phaseoloidin, and Iristectorigenin B.

#### 2.5.3. Physical Parameters-Alkaloid Correlation Analysis

A bidirectional O2PLS analysis was performed to examine the relationship between physical parameters and the relative abundance of alkaloid compounds, providing further insight into their correlation (Figure 7c). This approach aimed to identify the primary alkaloid compounds driving the association and display the top 10 compounds ranked by correlation. The results showed that the model parameters R^2^X and R^2^Y were 0.999 and 0.945, respectively, indicating a highly reliable model. The primary alkaloid components driving variations in physical parameters were identified as Laudanidine, Evoxine, Heliosupine N-oxide, Cropodine, Monocrotaline N-Oxide, Sceleratine N-oxide, Zinnimidine, Callimorphine, Crotafoline, and 1-(3, 4-Dimethoxycinnamoyl) piperidine.

### 2.6. Transcriptomics Analysis

#### 2.6.1. Differential Gene Identification

Based on the untreated group as control and MCF-7 cells exposed to three concentrations (0.3125 mg/mL, 0.625 mg/mL, and 1.25 mg/mL) of herbal extracts as experimental conditions, differential gene expression was analyzed using the FC-t algorithm. Applying thresholds of *p* < 0.05 and |FC| > 1.5, differentially expressed genes (DEGs) were identified by comparing transcript levels before and after treatment in both combined (HJD-co) and single (HJD-sin) decoction groups across low, medium, and high doses.

**Figure 8:**
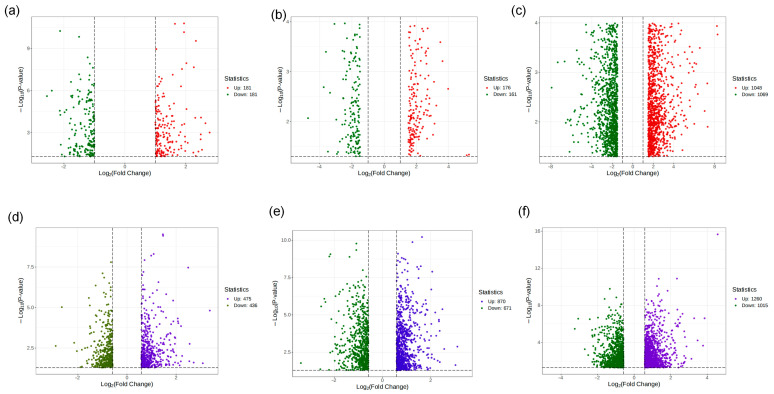
Differential gene analysis results between combined decoction and single decoction treatments. (**a**) Combined decoction low-concentration treatment group; (**b**) combined decoction medium-concentration treatment group; (**c**) combined decoction high-concentration treatment group; (**d**) single decoction low-concentration treatment group; (**e**) Single decoction medium-concentration treatment group; (**f**) single decoction high-concentration treatment group.

The number of DEGs in the combined decoction groups was 362 (low), 357 (medium), and 2117 (high), whereas the single decoction groups showed 911 (low), 1531 (medium), and 2275 (high) DEGs, respectively (Figure 8). These results reveal pronounced differences in gene expression patterns between the two decoction methods at each concentration level.

#### 2.6.2. GO Enrichment Analysis

**Figure 9:**
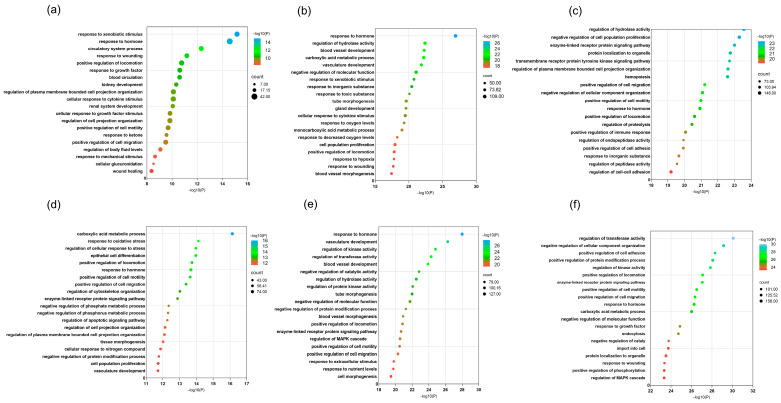
GO enrichment analysis results for combined decoction and single decoction at different concentrations. (**a**) GO enrichment analysis results for combined decoction at low concentration; (**b**) GO enrichment analysis results for combined decoction at medium concentration; (**c**) GO enrichment analysis results for combined decoction at high concentration; (**d**) GO enrichment analysis results for single decoction at low concentration; (**e**) GO enrichment analysis results for single decoction at medium concentration; (**f**) GO enrichment analysis results for single decoction at high concentration. Dot size represents the number of genes in each entry, and dot color indicates the enrichment level (*p*-value), ranging from blue to red, with deeper red showing stronger enrichment.

To elucidate the biological roles of each formulation and uncover the processes they influence, enrichment analysis was conducted on the differentially expressed genes using biological pathway (BP) terms from the Metascape database (https://metascape.org/gp, accessed on 6 June 2024). The top 20 most significantly enriched biological processes, ranked by *p*-value, are visualized in Figure 9.

Analysis revealed that the combined decoction primarily modulates processes including response to hormones, regulation of hydrolase activity, reaction to xenobiotic stimuli, wound healing, enhanced locomotion, organization of plasma membrane projections, promotion of cell migration, response to inorganic substances, and cytokine-mediated cellular signaling. In contrast, the single-ingredient decoction was mainly associated with carboxylic acid metabolism, vascular development, regulation of kinase and transferase activity, enzyme-linked receptor signaling, suppression of protein modification processes, negative regulation of molecular function, MAPK cascade control, and increased cell motility.

#### 2.6.3. KEGG Enrichment Analysis

**Figure 10:**
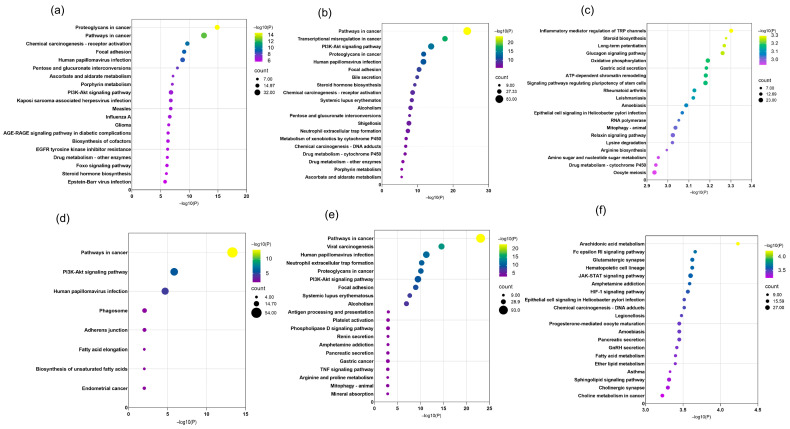
KEGG enrichment analysis results for combined decoction and single decoction at different concentrations. (**a**) KEGG enrichment analysis results for combined decoction at low concentration; (**b**) KEGG enrichment analysis results for combined decoction at medium concentration; (**c**) KEGG enrichment analysis results for combined decoction at high concentration; (**d**) KEGG enrichment analysis results for single decoction at low concentration; (**e**) KEGG enrichment analysis results for single decoction at medium concentration; (**f**) KEGG enrichment analysis results for single decoction at high concentration. Dot size represents the number of genes in each entry, and dot color indicates the enrichment level (*p*-value) ranging from blue to red, with deeper red showing stronger enrichment.

Using the Metascape platform, pathway annotation and functional analysis were conducted on the identified differentially expressed genes to elucidate key metabolic and signaling pathways involved. The top 20 signaling pathways ranked by significance (smallest *p*-values) were selected for visualization (Figure 10).

KEGG enrichment analysis revealed that in the combined decoction of HJD, the majority of genes were associated with pathways such as proteoglycans in cancer, pathways in cancer, PI3K-Akt signaling, chemical carcinogenesis—receptor activation, focal adhesion, human papillomavirus infection, pentose and glucuronate interconversions, ascorbate and aldarate metabolism, porphyrin metabolism, and drug metabolism—cytochrome P450. In contrast, the single-ingredient decoction primarily involved genes enriched in pathways in cancer, PI3K-Akt signaling, human papillomavirus infection, amphetamine addiction, and pancreatic secretion.

## 3. Discussion

This study identified clear differences in the physicochemical properties of HJD prepared by combined and single-herb decoction methods. The combined decoction showed higher conductivity, total dissolved solids, and salinity, attributed to a co-solvent effect generated by interactions among components during preparation. Lipophilic compounds with low water solubility show improved dispersion or dissolution when aided by components such as saponins, increasing their overall solubility. Intermolecular interactions occur among different chemical species, and under heating, oppositely charged ions or molecules, such as alkaloids with organic acids or flavonoids, form macromolecular complexes that may precipitate. Residual plant materials like cellulose also adsorb portions of active compounds.

Water-soluble polymers such as starch, proteins, polysaccharides, and gums dissolve during co-decoction, increasing medium viscosity and forming stable colloidal suspensions that encapsulate and stabilize hydrophobic molecules, resulting in uniform dispersion and distinct physical properties of the combined decoction [44,45]. In separately prepared herbs, each component dissolves independently without synergistic interaction, and poorly soluble substances often remain partially undissolved due to limited solubility [46].

When a single herb is decocted, its chemical composition remains restricted without complementary compounds for complex formation, and any precipitation mainly results from solubility differences among its own constituents. Limited interactions may occur after mixing separately prepared decoctions, but these are weaker than those formed during co-decoction, producing a simpler and less stable system [47,48]. Without the colloidal structure generated in co-decoction, the single-herb mixture shows reduced physical stability and a stronger tendency for phase separation over time.

When herbs are decocted together in traditional Chinese medicine, their constituents undergo interactions such as complexation, condensation, esterification, and Maillard reactions under heat, generating new compounds not found in separately prepared extracts, which may contribute to therapeutic effects [49]. Berberine from *Coptis chinensis Franch* is a quaternary ammonium alkaloid with a positive charge, while the main components of *Scutellaria baicalensis Georgi* (baicalin) and *Gardenia jasminoides Ellis* (geniposide) contain negatively charged carboxyl or phenolic hydroxyl groups. During co-decoction, these opposite charges promote the formation of berberine–baicalin and berberine–geniposide complexes stabilized by ionic and hydrogen bonds, a process that occurs far less when herbs are decocted separately [50]. Although the concentration of free berberine decreases, new chemical entities such as precipitate complexes and condensation products appear, which alter the pharmacological and pharmacokinetic properties of the formula and may allow gradual gastrointestinal breakdown, sustained release, reduced toxicity, or targeted therapeutic action.

The mixture produced by single decoction contains higher amounts of free berberine and baicalin but lacks the complex structures formed during co-decoction. Berberine interacts with baicalin and wogonin through electrostatic and hydrophobic forces, resulting in the spontaneous formation of self-assembled nanoparticles that display stronger antibacterial activity than their individual components [51]. Geniposide, an active compound from gardenia fruit, significantly increases the oral bioavailability of berberine by inhibiting its metabolic degradation, producing synergistic therapeutic effects and enhancing intestinal absorption [52]. During co-decoction, micro- and nanoscale aggregates form, improving the bioavailability of poorly soluble compounds such as baicalin. The concentrations of marker constituents in combined decoctions differ from the arithmetic sum of individual extracts because precipitation, adsorption, and the formation of new chemical entities alter the final composition.

Further research is needed to clarify the efficacy differences between the combined and separate decoction methods of Huanglian Jiedu decoction. Although this study identified significant variations in physicochemical and compositional characteristics between the two preparations, the mechanisms underlying these differences remain unclear, and it is uncertain whether such patterns apply broadly to other Chinese herbal formulas.

In conclusion, this work documents the physicochemical variations and interrelationships in HJD preparations, offering new perspectives for future studies examining the characteristic differences between traditional combined and modern separate decoction techniques for herbal medicines.

## 4. Materials and Methods

### 4.1. Materials

*Coptis chinensis Franch*, *Scutellaria baicalensis Georgi*, *Phellodendron chinense Schneid*, and *Gardenia jasminoides Ellis* were all purchased from Jilin Province Beiyao Herbal Processing Co., Ltd. (Changchun, China), and authenticated by Professor Weng Lili, Head of the Department of Chinese Materia Medica Identification and Processing at the School of Pharmacy, Changchun University of Chinese Medicine. Huanglian is the dried rhizome of *Coptis chinensis Franch*, a plant of the Ranunculaceae family. Huangbo is the dried bark of *Phellodendron chinensis Schneid*, a plant of the Rutaceae family. Huangqin is the dried root of *Scutellaria baicalensis Georgi*, a plant of the Lamiaceae family. Zhizi is the dried mature fruit of *Gardenia jasminoides Ellis*, a plant of the Rubiaceae family. Coptisine chloride (B21438, 98%), Epiberberine (B20108, 98%), Palmatine chloride (B21433, 98%), Berberine hydrochloride (B21449, 98%), Jatrorrhizine hydrochloride (B21451, 98%), Phellodendrine chloride (B21437, 98%), Baicalin (B20570, 98%), and Geniposide (B21661, 98%) were purchased from Yuanye Biotechnology Co., Ltd. (Shanghai, China); Agilent 1260 HPLC (Santa Clara, CA, USA), Q Exactive HFX mass spectrometer (Thermo, Waltham, MA, USA), Vanquish Ultra-High Pressure Liquid Chromatograph (Thermo), Methanol was purchased from Fisher (Waltham, MA, USA). Acetonitrile, formic acid, and isopropyl alcohol were purchased from Amper Company (Haverhill, UK). All other chemical reagents and solvents were of analytical grade.

### 4.2. Methods

#### 4.2.1. Combined Decoction and Single Decoction Preparation Processes

According to the renowned Chinese medical formula compendium *Wai Tai Mi Yao* compiled by Tao Wang, the preparation method for Huanglian Jiedu decoction is as follows: “Three liang of *Coptis chinensis Franch*, two liang each of *Scutellaria baicalensis Georgi* and *Phellodendron chinensis Schneid*, fourteen *Gardenia jasminoides Ellis* (split).” Converted to modern measurements, this equates to the following: Combined decoction: Prepare the herbs using traditional decoction methods. Take 41.4 g of *Coptis chinensis Franch*, 27.6 g of *Scutellaria baicalensis Georgi*, 27.6 g of *Phellodendron chinense Schneid*, 15 g *Gardenia jasminoides Ellis*, add 1200 mL water, bring to a boil over high heat (2200 W), then simmer over low heat (800 W) for 40 min. Immediately filter while hot using two layers of 200-mesh gauze to obtain 400 mL of decoction. Freeze-dry for 72 h to yield the combined decoction freeze-dried powder.

Single decoction: Take 41.4 g of *Coptis chinensis Franch*, 27.6 g of *Scutellaria baicalensis Georgi*, 27.6 g of *Phellodendron chinense Schneid*, and 15 g of *Gardenia jasminoides Ellis*. Add water separately: 414 mL, 276 mL, 276 mL, and 15 mL. Heat vigorously (2200 W) until boiling, then simmer gently (800 W) for 40 min. Immediately filter while hot using two layers of 200-mesh gauze. Freeze-dry for 72 h to obtain the single-decoction freeze-dried powder.

Repeat the above steps in parallel three times to obtain freeze-dried powder from the combined decoction at different cooking times and freeze-dried powder from the single decoction.

#### 4.2.2. Establishment of an HPLC Method

##### Preparation of Mixed Reference Solution

Precisely weigh appropriate amounts of Epiberberine, Jatrorrhizine hydrochloride, Coptisine chloride, Berberine hydrochloride, Phellodendrine chloride, Palmatine chloride, Geniposide, and Baicalin reference standards, dissolve in methanol to prepare a mixed reference solution with mass concentrations of 10.9, 21.2, 17.3, 13.3, 560, 11.9, 23.2, and 15.2 μg/mL, respectively.

##### Preparation of Test Solution

Combined decoction: Precisely weigh 0.2 g of freeze-dried powder. Precisely add 50 mL of methanol, weigh the mass, subject to ultrasonic treatment for 30 min (250 W power, 70 Hz frequency), weigh the sample again, add an appropriate amount of methanol to compensate for weight loss, and filter using filter paper. Add methanol to make up the volume to a 10 mL volumetric flask, and shake well to yield the combined decoction test solution.

Single decoction: Extracts of *Coptis chinensis Franch*, *Scutellaria baicalensis Georgi*, *Phellodendron chinense Schneid*, and *Gardenia jasminoides Ellis* were prepared at a crude drug ratio of 3:2:2:1. Take 0.075 g *Coptis chinensis Franch*, 0.05 g *Scutellaria baicalensis Georgi*, 0.05 g *Phellodendron chinense Schneid*, and 0.025 g *Gardenia jasminoides Ellis*, and mix thoroughly, Precisely add 50 mL of methanol, weigh the mixture, subject to ultrasonic treatment for 30 min (250 W power, 70 Hz frequency), weigh again, replenish the loss in weight with methanol, filter through filter paper, dilute to 10 mL in a volumetric flask with methanol, and shake well to yield the single decoction test solution.

##### Chromatographic Conditions

The column is a ZORBAX Eclipse Plus C18 (250 nm × 4.6 mm, 5 μm); the mobile phase consists of methanol (A) and 0.1% phosphoric acid in water. Gradient elution: 0–20 min, 20–30% A; 20–50 min, 30–40% A; 50–60 min, 40–50% A; 60–70 min, 50–60% A; 70–80 min, 60–80% A; 80–81 min, 80–20% A; and 81–86 min, 20% A. Flow rate: 0.6 mL/min. Detection wavelength: 238 nm. Column temperature: 35 °C. Injection volume: 5 μL.

#### 4.2.3. Development of a UPLC-MS/MS Method

##### Sample Extraction

Accurately weigh 100 mg each of the combined decoction and single decoction samples into centrifuge tubes. Add 1 mL of extraction solvent (water/acetonitrile/isopropanol, 1:1:1, *v*/*v*/*v*), vortex for 60 s, perform low-temperature ultrasonic extraction for 30 min, centrifuge at 12,000 rpm for 10 min at 4 °C, and collect the supernatant. Allow proteins to precipitate at −20 °C for 1 h, then centrifuge again at 12,000 rpm for 10 min at 4 °C, resuspend in 200 μL 50% acetonitrile solution, vortex, centrifuge at 14,000 rpm for 15 min at 4 °C, and collect the supernatant for instrument analysis.

##### Instrument Parameters

Liquid Chromatography Parameters Column: Waters HSS T3 (100 × 2.1 mm, 1.8 μm); Mobile Phase: Phase A is ultrapure water solution (containing 0.1% formic acid), Phase B is acetonitrile solution (containing 0.1% formic acid); flow rate: 0.3 mL/min; column temperature: 40 °C; injection volume: 2 μL; elution gradient: 0 min A/B (100:0, *v*/*v*), 1 min A/B (100:0, *v*/*v*), 12 min A/B (5:95, *v*/*v*), 13 min A/B (5:95, *v*/*v*), 13.1 min A/B (100:0, *v*/*v*), and 17 min A/B (100:0, *v*/*v*) [53]. Throughout the entire analysis process, the sample is maintained at a constant temperature of 4 °C within the autosampler.

Mass Spectrometry Conditions: Primary and secondary mass spectra were acquired using a Thermo Q-Exactive HFX high-resolution mass spectrometry system (Thermo, Waltham, MA, USA). The instrument used an electrospray ionization source with a sheath gas flow of 40 arb, auxiliary gas flow of 10 arb, ion spray voltage of +3000 V and −2800 V, source temperature of 350 °C, and ion transfer tube temperature of 320 °C, operating in Full-MS-ddMS2 scanning mode under both positive and negative ion conditions. The primary mass spectrum scan range was 70–1050 Da, with a primary resolution of 70,000 and a secondary resolution of 17,500.

#### 4.2.4. Physical Parameter Testing

Combine the HJD combined decoction and single-decoction samples with an equal volume of purified water for dissolution. Use a conductivity meter to measure TDS, salinity, conductivity, and resistivity. After calibrating the instrument, immerse the electrode tip below the surface of the sample solution and take readings to obtain the corresponding values.

#### 4.2.5. Phase Separation

Centrifuge the combined decoction from Section Multivariate Statistical Analysis and the single decoction combined solution at 10,000 rpm for 30 min to obtain precipitate fraction I. Take the supernatant and dialyze it for 30 min at 37 °C in a water bath using a dialysis bag with a molecular weight cut-off of 3500. Centrifuge at 5000 rpm for 30 min. Repeat this dialysis–centrifugation process three times. After three dialysis cycles, combine the sediment fractions obtained from dialysis centrifugation to form the suspension phase II. The dialysis bag contains the nano-phase fraction III. The decoction is thus separated into three phases: nano-phase, suspension phase, and sediment phase.

#### 4.2.6. SEM Observation

Prepare freeze-dried powders from combined and single-boiled extracts. Apply gold coating to enhance conductivity. Start the SEM system and wait for the vacuum system to reach the appropriate level. Place the sample on the stage, adjust the focus and contrast, and optimize scanning parameters, including scanning speed and scanning area, to obtain the desired image details.

#### 4.2.7. Transcriptome Sequencing

The combined decoction and single decoction water extracts served as positive controls. Using human breast cancer cells MCF-7 (obtained from Cyagen, Santa Clara, CA, USA) as a model, the 24 h IC50 concentration of the drug was determined to be 1.25 mg/mL. Log-phase MCF-7 cells (1 × 10^4^ cells/100 μL) were seeded into 96-well plates. After 24 h incubation, cells were treated with drug concentrations of 1.25 mg/mL, 0.625 mg/mL, and 0.3125 mg/mL, with untreated cells serving as the negative control. After another 24 h of incubation, total RNA was extracted and analyzed for transcriptomic changes using the High-Throughput Gene Detection System (HISTAG). Each concentration was tested in three biological replicates.

## 5. Conclusions

Observations show that Huanglian Jiedu decoction (HJD) prepared by combined decoction has a darker color and higher turbidity than the single-decoction mixture, often with visible sediment. HPLC analysis showed that the combined decoction contained lower levels of free berberine than the single-herb mixture. UPLC-MS/MS identified flavonoids and alkaloids as the main components distinguishing the two preparations. Physical measurements indicated a higher content of water-insoluble substances in the combined decoction, and phase separation analysis confirmed the presence of nano-aggregates absent in the single decoction. Transcriptome analysis showed distinct gene expression patterns among different concentrations and identified the major biological pathways affected.

These results indicate essential physicochemical differences between the two preparation methods, showing that the combined decoction forms an integrated system in which solubilization, precipitation, complexation, and the formation of new compounds generate a distinct material basis that provides a theoretical foundation for subsequent pharmacological research and clinical applications.

## Data Availability

The original contributions presented in this study are included in the article. Further inquiries can be directed to the corresponding author.

## Abbreviations

HJD	Huanglian Jiedu decoction
HPLC	High-Performance Liquid Chromatography
UPLC-MS/MS	Ultra-High Performance Liquid Chromatography-Mass Spectrometry
NPS	Nano-phase state
PP	Precipitate Phase
